# Cognition and Mood-Related Behaviors in *L3mbtl1* Null Mutant Mice

**DOI:** 10.1371/journal.pone.0121252

**Published:** 2015-04-07

**Authors:** Erica Y. Shen, Yan Jiang, Wenjie Mao, Kensuke Futai, Hanno Hock, Schahram Akbarian

**Affiliations:** 1 Department of Psychiatry, Friedman Brain Institute, Icahn School of Medicine at Mount Sinai, New York, New York, 10029, United States of America; 2 Brudnick Neuropsychiatric Research Institute, University of Massachusetts Medical School, Worcester, Massachusetts, 01604, United States of America; 3 Cancer Center and Center for Regenerative Medicine, Massachusetts General Hospital, Boston, Massachusetts, 02114, United States of America; University of Tokyo, JAPAN

## Abstract

Alterations in histone lysine methylation and epigenetic regulators of gene expression could play a role in the neurobiology and treatment of patients diagnosed with mood spectrum disorder, including depression and anxiety. Mutations and altered expression of various lysine methyltransferases (KMTs) and demethylases (KDMs) have been linked to changes in motivational and emotional behaviors in preclinical model systems. However, it is not known whether regulators operating downstream of histone lysine methylation could affect mood-related behavior. *Malignant Brain Tumor* (MBT) domain ‘chromatin reader’ proteins bind to methylated histone lysine residues and associate with chromatin remodeling complexes to facilitate or repress gene expression. MBT proteins, including the founding member, *L3mbtl1*, maintain high levels of expression in neurons of the mature brain. Here, we exposed *L3mbtl1* null mutant mice to a wide range of tests exploring cognition and mood-relevant behaviors at baseline and in the context of social isolation, as a stressor to elicit depression-related behavior in susceptible mice. *L3mbtl1* loss-of-function was associated with significant decreases in depression and and anxiety in some of the behavioral paradigms. This was not associated with a more generalized neurological dysfunction because cognition and memory remained unaltered in comparison to controls. These findings warrant further investigations on the role of MBT chromatin reader proteins in the context of emotional and affective behaviors.

## Introduction

Mood spectrum disorders, including depression and various anxiety disorders [1], cause significant morbidity and mortality with at least 40% of subjects only partially responding to currently available pharmacological treatments that primarily target monoamine metabolism and reuptake [2]. Among the novel therapeutic avenues currently pursued in preclinical studies are epigenetic mechanisms, affecting chromatin structure and function as well as gene expression in response to diverse stimuli [3,4]. Various regulators of post-translational histone modifications, including enzymes targeting histone lysine residues for deacetylation, methylation, and demethylation, recently emerged as promising starting points for novel epigenetic drug targets in the treatment of depression and anxiety based on work in animal models and postmortem brain of subjects diagnosed with major mood disorders and other psychiatric disease [5–13]. Much less is known the molecular machinery operating downstream of the histone modifications implicated in neuronal plasticity and behavior, such as ‘chromatin-readers’ [14].

*Malignant Brain Tumor* (MBT) domain chromatin reader proteins bind to methylated histone lysine residues and associate with chromatin remodeling complexes. These include typical and atypical Polycomb repressor complexes that control expression of genes with essential roles in cell cycle regulation and early development [15,16]. Several MBT proteins, including the founding member, *L3mbtl1*, maintain very high levels of expression in mature brain with broad, near-ubiquitous expression in fore-, mid- and hindbrain neurons [17]. This suggests that MBT proteins may serve yet unknown functions in terminally differentiated brain cells. Proper cell-type specific regulation of histone methylation landscapes is essential for normal brain function [18,19], and rare mono- or oligogenic forms of cognitive disease have been linked to mutations in histone lysine methyltransferase (*KMT*) and demethylase (*KDM*) genes. These broadly affect the epigenetic landscapes at sites of open or repressive chromatin [5,20–22]. Motivational and emotional behaviors have also been linked to histone lysine methylation. For example, *Kmt1a(Suv39h1)*-, *Kmt1c(G9a)*- and *Kmt1e(Setdb1)*-mediated repressive H3K9 methylation regulate amphetamine reward [23,24] and antidepressant-like behaviors in mice[25]. Furthermore, monoamine oxidase inhibitors exert powerful effects on mood and cognition primarily by elevating brain monoamine levels, but, intriguingly, some members of this class of drugs also interfere with lysine methylation pathways by blocking *Kdm1a/Lsd1* amine oxidase domain containing KDMs [26,27]. Therefore, exploration of regulators and readers of histone lysine methylation marks, such as the MBT family of genes, bears potential promise to discover novel treatment avenues for mood spectrum disorders, including anxiety and depression. However, there is very little information on neurological phenotypes after loss of specific MBT genes. Thus it remains unclear whether the MBT lysine methylation reader proteins are essential for mature brain function. Here, we provide a detailed account for a comprehensive behavioral assessment of adult *L3mbtl1* null mutant mice exposed to a wide range of behavioral tests at baseline and after single housing as a social isolation stressor eliciting depression-related behavior in susceptible mice [28].

## Methods

All animal work of this study has been approved by the Institutional Animal Care and Use Committee of the Icahn School of Medicine at Mount Sinai. Generation of *L3mbtl1-/-* null mutant line has been described previously [29]. Mice are held under specific pathogen-free conditions with food and water being supplied ad libitum in an animal facility with a reversed 12 h light/dark cycle (light off at 7:00 am) under constant conditions (21 ± 1°C; 60% humidity).

For the present study, male and female null mutant and wildtype mice, all from the same colony in a predominant C57Bl6/J background, back-crossed for at least 5 generations, and 3–6 months of age, were used. Furthermore, all mice used for the actual experiments were derived from heterozygous (*L3mbtl1+/-*) breeder pairs. For all test conditions, the male:female ratio was approximately 1:1, and each mutant animal was analyzed in parallel to one or two sex-matched wildtype control of the same age ± 2 weeks. All animals had access to food and water ad libidum. All animals (including those exposed to social isolation were monitored at least 2–3 times each week to assess well-being and to monitor for any signs of distress.

### Anxiety assays

#### Open field locomotion test

This test was monitored in test chambers using a photocell-beam-based computer detecting system (OmniTech Electronics. Inc). The apparatus consisted of an arena (40 x 40 cm) surrounded by 40 cm high walls made from clear plastic. Animals were introduced into the corner of the test chamber and allowed free exploration for 20 min individually under standard room lighting conditions. The beam breaks were recorded every 5 min to evaluate the spontaneous locomotor activity. Total distance (indicative of locomotor activity) and the duration of time the animals spent in the central and peripheral areas were also calculated.

#### The light/dark box test

This test was performed in the open field arena with a black box insert (20 cm L x 20 cm W x 40 cm H), dividing the arena into dark and light components connected by a small hole. Animals were introduced into the dark chamber and allowed free exploration for 10 min. The duration of time spent in the dark and light chambers were calculated.

#### Elevated Plus Maze test

The elevated plus maze (Med Associates Inc) contained a center square (6cm x 6cm), two open arms, and two closed arms (measuring 35cm x 6cm each). The closed arms were enclosed by black polypropylene walls measuring 20 cm in hight. Mice were placed in the center square facing one of the closed arms. Time spent in each arm was recorded and scored by the EthoVision video tracking system. A decrease in time spent in the open arms reflects a state of increased anxiety.

### Behavioral despair/depression assays

#### Tail suspension test

Animals were suspended with duct tape by the tail. Animals showing tail climbing behaviors were removed from the statistical analysis. The whole test was videotaped for 5 minutes. Latency to first freezing and the time spent immobile was evaluated by EthoVision software (Noldus, Wageningen, The Netherlands).

#### Forced swim test

Animals were placed into a 4 L Pyrex beaker (13 cm diameter, 24 cm height) filled with 17cm of 22°C water. The whole test was videotaped for 5 minutes. Latency to first freezing and time spent immobile was evaluated by EthoVision software.

### Cognition and working memory assays

#### Contextual fear conditioning test

Contextual fear conditioning was conducted in a fear conditioning chamber from Med Associates Inc. Mice were placed in the chamber for a 7 min training session during which time 3 shocks were delivered. The percentage of time freezing before the first shock (baseline) and after each shock was measured. After 24 hours, mice were returned to the same chamber for a 3-min retrieval testing and percentage of time freezing was measured.

#### Radial arm maze test

The radial arm maze test was conducted to measure working memory. The apparatus consisted of 8 arms (5 x 50 cm; 30 cm high walls), which were assembled in a radial manner around a circular starting platform. Mice were tested for 4 consecutive days in the apparatus. On each day mice were placed onto the starting platform and were free to enter the arms. The test was continued until all eight arms had been visited at least once. A mistake was defined as a repeated entry to an already visited arm before each of the eight arms was visited. Total mistakes during each session were measured. Because this protocol measures spontaneous alternations, food deprivation in conjunction with baited arms was not used.

#### Dopamine-mediated locomotion test

For this test, mice were placed into an open-field chamber (OmniTech Electronics. Inc) with their baseline activity recorded for 30 min, then injected with 0.9% saline and locomotor activity recorded for 30 additional min. Finally, mice were injected with the D1 receptor agonist SKF81297 at doses of 0.5 and 1 mg/kg body weight and recorded for a final 90 min.

### Social Isolation Stress Test

#### Single housing test

All animals were consistently group-housed during the entire pre- and post-weaning period, until the beginning of postnatal week four when a subset were switched to single-housed. After a 3-month single-housing period, behavioral testing on anxiety, depression, working memory and locomotion were conducted in single-housed mice, in parallel to group-housed mice.

### Statistical analyses

Two-tailed unpaired t-test was applied to test for significance of differences between *L3mbtl1-/-* and wildtype controls for body weight, behavioral despair (tail suspension and forced swim) and anxiety (elevated plus maze, light/dark box) test. For comparison of group- vs. single-housed animals (open field, behavioral despair, radial arm maze), two-way ANOVA followed by post-hoc t-test was applied. For monitoring locomotor activity after acute treatment with D1 agonist (SKF81297), repeated measures ANOVA was applied, followed by post-hoc t-test when indicated.

## Results

**Fig 1:**
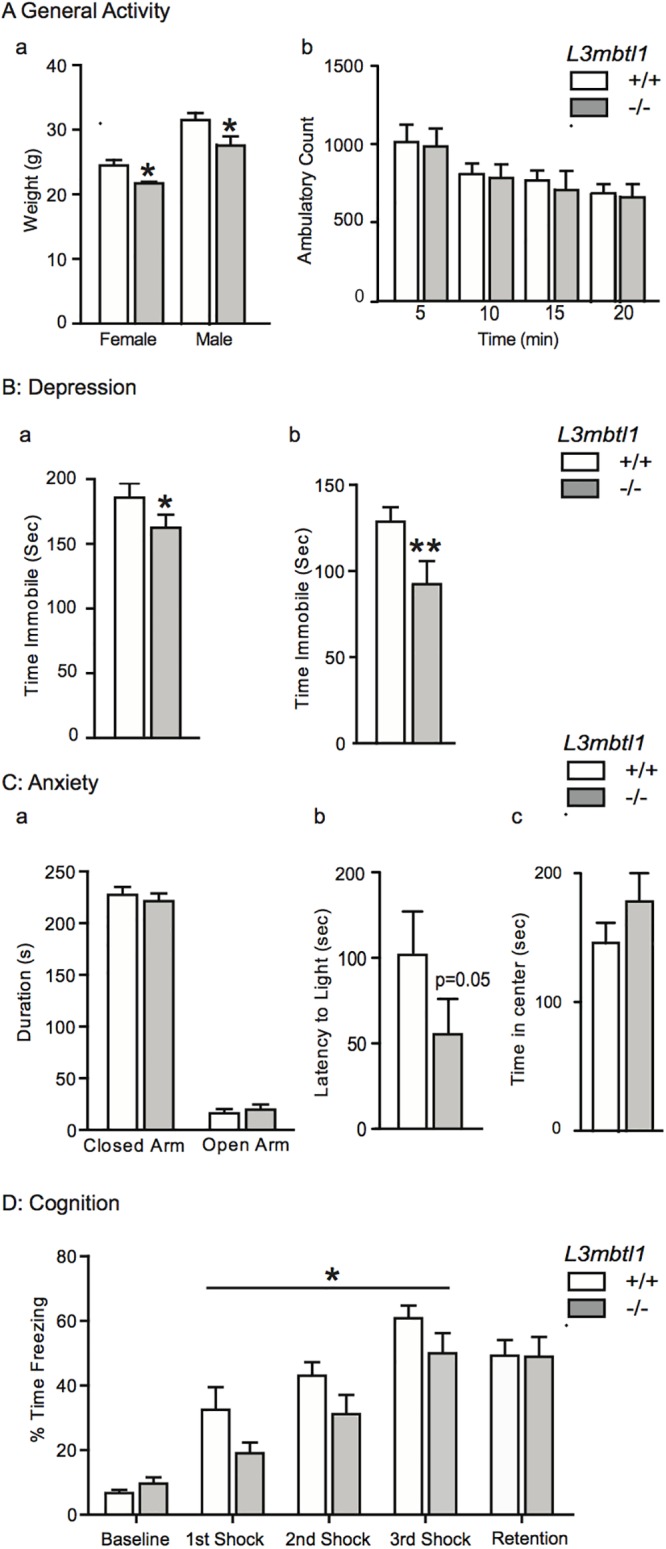
Behavioral summary of *L3mbtl1* null mutant and control mice at baseline. (**A**) General activity, (a) body weight and (b) locomotion. (**B**) Depression, (a) forced swim and (b) tail suspension (N = 25-28/genotype and test). (**C**) Anxiety, (a) elevated plus maze, (b) light-dark box, (c) open-field test (N = 17–21 genotype/test). (**D**) cognition, assessed by contextual fear conditioning (N = 12 *L3mbtl1-/-* and N = 17 *L3mbtl1+/+*). Animals used for general activity and depression assays were from a different batch than the animals used for anxiety assays. A third batch of animals was used for the fear conditioning assays. All animals are kept under baseline conditions (group housing). All data are shown as mean ± S.E.M., * P <0.05; independent-samples t test and, for fear conditioning assay, repeated measures ANOVA.

We reported previously that *L3mbtl1-/-* null mutant mice exhibit normal life expectancy, a grossly normal brain morphology and cytoarchitecture, and scorings in a locomotor coordination test that were indistinguishable from controls[29]. Notably, however, both male and female *L3mbtl1-/-* show a subtle but significant reduction in body weight at 3–6 months of age (approximately 10%) compared to gender- and age-matched controls, while overall locomotor activity was indistinguishable between mutants and controls (Fig 1A). This is likely important because a wide range of genetically engineered mice with altered baseline body weight frequently show robust changes in motivational and affective behaviors. Such abnormalities were, for example, observed in mouse lines carrying loss-of-function mutations for specific chromatin regulators like the Rett syndrome gene *Methyl-CpG-binding protein 2 (Mecp2)*[30–32], and the transcription factor *Circadian locomotor cycles output kaput (Clock)*[33,34]. Therefore, we asked whether depression and anxiety-related behaviors are altered in *L3mbtl1-/-* mice that were kept under normal ‘non-stressed’ group-housing conditions (2–3 sex-matched littermates as cage mates after weaning).

We first applied two widely used depression-relevant tests, the forced swim and tail suspension tests, which are considered behavioral despair paradigms[35], as a measure for depression[36] and, importantly, as measure for the effectiveness of antidepressant drugs [37]. Interestingly, *L3mblt1-/-* mice showed a significant, approximately 30% decrease in immobility in the tail suspension test (Fig 1B, panel a). Likewise, mutant mice showed a subtle, approximately 15% decrease in immobility in the forced swim test, in comparison to *L3mbtl1+/+* controls (Fig 1B, panel b). Both of these tests suggested that *L3mbtl1* loss reduced behavioral patterns associated with depression.

Because depression and anxiety spectrum disorders show considerable overlap in terms of diathesis and underlying neurobiology [1,38,39], we next explored the performance of *L3mbtl1-/-* mice in several anxiety-relevant paradigms. We applied the elevated plus maze, which is based on the animals aversion of open spaces, resulting in avoidance of open areas and confining movements to enclosed spaces [40]. However, both *L3mbtl1* null mutant and control animals showed a similar strong preference for the closed as opposed to the open arms in the elevated plus maze (Fig 1C, panel a).

Next, we tested in the animals in the light-dark box test [41]. The test’s basic measure is the animal’s preference for dark, enclosed places over bright, exposed places. The time spent in the light box, and the related exploratory behaviors, inform about the level of anxiety. The *L3mbtl1-/-* mice showed a significant decrease in latency to enter the bright chamber in the light-dark box test [41], indicating decreased anxiety (Fig 1C, panel b). We then applied a third anxiety paradigm, the open field test [42]. This test measures emotionality and anxiety in rodents, including the time spent in the (anxiogenic) open center (as opposed to the for rodents less anxiogenic space along the apparatus’ walls), and the general activity in the first few minutes after being place into the apparatus [43]. Of note, the *L3mbtl1 -/-* mice spent more time in the center by a 15% difference across the 20 min test period (Fig 1C, panel c). The latter result did not reach statistical significance but is consistent with the notion that anxiety was reduced. Taken together, these findings suggest that *L3mbtl1* disruption is followed by subtle anxiolytic and antidepressant-like effects that become manifest in some but not all depression- and anxiety-related paradigms. To further examine whether these phenotypes in *L3mbtl1-/-* null mice are representative of broader changes in cognition and behavior, we exposed animals to a contextual fear paradigm exploring retrieval-dependent freezing behavior 24 hours after exposure to electrical foot shocks in the same test chamber. This test essentially explores cognitive behavioral plasticity resulting from coupling a neutral stimulus with an aversive stimulus, and is heavily dependent on neural circuitry of the amygdala, while the role of the hippocampus is less prominent for this type of learning[44]. However, retrieval was indistinguishable between mutant and controls (Fig 1D), suggesting normal long-term memory function in *L3mbtl*1 null mice. Nevertheless, mutant mice showed a consistent, 25–40% decline in freezing time across all three training days, which was significant (P <0.05, repeated measures ANOVA) (Fig 1D).

**Fig 2:**
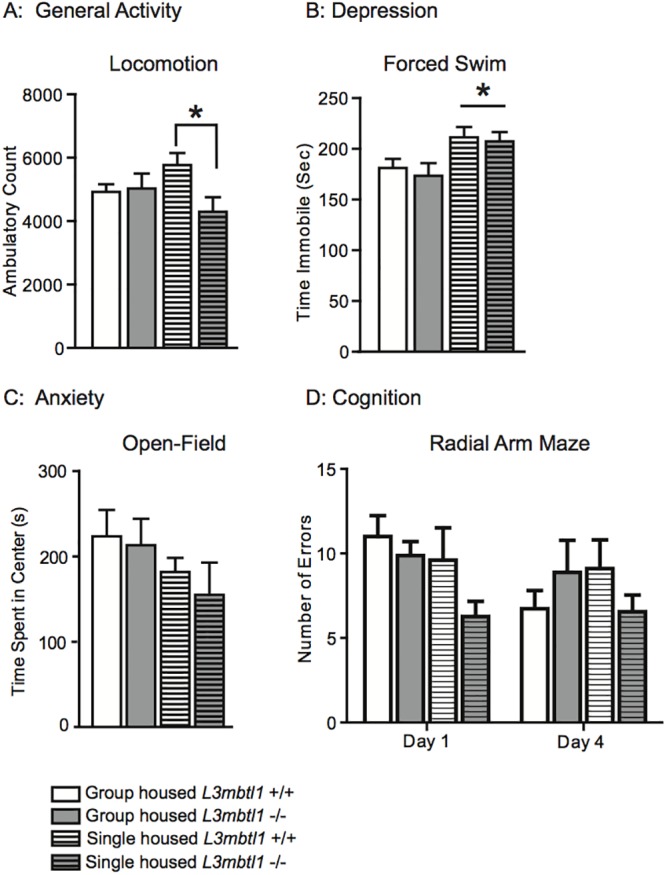
Behavioral summary in group housed versus single housed (3 months of social isolation) *L3mbtl1* null mutant and wildtype mice. The figure summarizes the following tests in group and single housed mice, as indicated (**A**) General activity/locomotion. (**B**) Depression/ forced swim. (**C**) Anxiety/open field test. (N = 8 group housed *L3mbtl1-/-*, N = 10 group housed *L3mbtl1+/+*, N = 10 single housed *L3mbtl1-/-*, N = 10 single housed *L3mbtl1+/+*). (**D**) Cognition/radial arm maze (N = 8 group housed *L3mbtl1-/-*, N = 11 group housed *L3mbtl1+/+*, N = 11 single housed *L3mbtl1-/-*, N = 10 single housed *L3mbtl1+/+*). Behavioral assays were conducted in the order from the least to the most stressful, and thus (i) activity/locomotion, followed by (ii) radial arm maze, followed by (iii) anxiety/open field, and (iv) depression / forced swim as the final test. All data shown as mean ± S.E.M., * P <0.05; independent-samples t-test after two-way ANOVA: for (A) locomotion: F_(1,34)_ = 3.807, P = 0.05, Bonferroni post test P <0.05 for single housed *L3mbtl1-/-* vs. *L3mbtl1+/* +); for (B) forced swim: F_(1,34)_ = 10.93, P = 0.022, Bonferroni post test P <0.01 for single vs. group housed; for (C), (D) no significance. All data shown in Fig 2 are from batches of mice different from those shown in Fig 1.

Given that *L3mbtl1* mutant mice exhibit subtle alterations in anxiety-related behavior at baseline, we then asked whether cognitive and emotional functions show changes in the context of a stressor such as social isolation by housing mice individually. Such stressors elicit depression-related behaviors in susceptible mice [28]. To this end, another batch of mutant and controls (different from the animals used for the studies shown in Fig 1A–1D) were single- or group-housed after weaning, then at 3–4 months of age subjected to open field (anxiety) and forced swim (behavioral despair) and the eight-arm radial arm maze for working memory. In rodents, increased open field locomotion after a period of social isolation is thought to reflect increased levels of anxiety[45]. As expected, social isolation induced increased open field locomotion in *L3mbtl1+/+* animals, but this effect was completely lacking in socially isolated *L3mbtl1-/-*, or in group-housed mice (Fig 2A). The ~ 25–30% increase in locomotion after social isolation in *L3mbtl1+/+*, in comparison to null mutant mice, was significant (Fig 2A) and may indicate that loss of *L3mbtl1* renders mice less susceptible to increased anxiety after certain types of stress. However, single housing induced both in *L3mbtl1* null and wildtype mice an ~ 25% increase in despair-induced immobility in the forced swim paradigm compared to group-housed animals (Fig 2B). Furthermore, housing conditions did not affect performance in the open field test (Fig 2C). Thus, *L3mbtl1-/-* showed a specific resilience to social isolation in the open field, but not in other anxiety- and depression-related tests.

Next, we measured spatial working memory as measured by repeat entries (‘errors’) in the 8-arm radial maze in group- and single-housed *L3mbtl1+/+* and *L3mblt1-/-* mice. Importantly, working memory, defined as a limited capacity storage system involved in information processing over a short time period [46], is associated with a variety of emotional aspects and sensitive to stress and depression [47]. Therefore, we hypothesized that *L3mbtl1-/-* mice may perform differently from controls in such type of cognitive task. In the 8-arm radial maze paradigm, applied once daily for 4 days, single-housed *L3mbtl1-/-* tended to outperform single-housed controls but these differences did not reach the level of significance and no consistent genotype effect was observed overall (Fig 2D).

**Fig 3:**
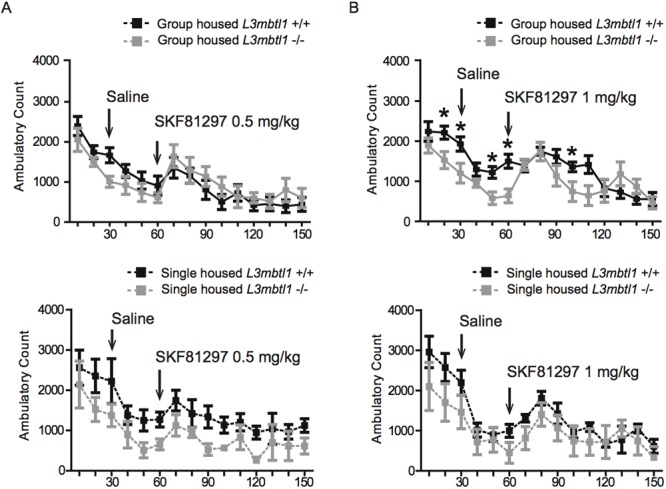
Locomotor effects of D1 agonist SKF81297. The figure summarizes SKF81297-induced locomotion at (**A**) 0.5mg/kg and (**B**) 1mg/kg i.p., administered 60 min after placing mice into the open field apparatus and 30 min after a saline control injection. Assays were conducted in group and single housed animals, as indicated (N = 5 group housed *L3mbtl1-/-*, N = 9 group housed *L3mbtl1+/+)* and single-housed animals N = 4 single housed *L3mbtl1-/-*, N = 5 single housed *L3mbtl1+/+*). All data are shown as mean ± S.E.M. (B) repeated measures ANOVA F_(1,14)_ = 3.203 with time point T _60 min_ Bonferroni post test P <0.05, other time timepoints *, P <0.05 by independent sample t-test. All data in Fig 3 are from experimentally naive mice.

Dopaminergic activity, including dopamine D1-receptor mediated signaling, is also sensitive to stressful exposure and potentially altered in some of the depression-relevant paradigms[48]. Therefore, in our final set of experiments, using naive animals that had not been included in any of our behavioral assays described above, we explored the effects of a dopaminergic drug on group- versus single housing on locomotor activity in *L3mbtl1-/-* and *L3mbtl1+/+* mice, using the open field apparatus. We treated mice (after 30 min with saline and, after another period of 30 min) with SKF81297, a dopamine D1 receptor agonist and mild stimulant drug [49], at doses of first 0.5 mg/kg and subsequently 1 mg/kg. Animals had one week of rest before receiving the second dose. In this paradigm, *L3mbtl1-/-* mice showed subtle decreases in locomotor activity, while both mutant and wildtype animals showed increased locomotion after administration of the dopaminergic drug, as expected (Fig 3A and 3B). However, significant differences were limited to group-housed animals due to increased locomotion in wildtype animals at multiple time points before and after administration of the higher (1mg/kg) dose of SKF81297 (Fig 3B). We conclude that *L3mblt1-/-* mutant mice indeed show decreased locomotor activity for at least some of the time periods tested in the open field paradigm, but this is not associated with a generalized hyper- or hypo-responsiveness to dopamine D_1_ receptor agonists. These data would suggest that alterations in dopamine D_1_ signaling are unlikely to be a major factor in the subtle changes in mood and anxiety related behaviors in the *L3mbtl1* null mutant mice of the present study.

## Discussion

MBT-domain proteins, including L3MBTL1, bind to methylated lysine residues and regulate chromatin structure and function by mediating nucleosomal compaction and affecting the activity of chromatin remodeling complexes, such as Polycomb[50–52]. *L3mbtl1* is highly expressed in neurons of the adult mouse brain. As fine-tuning and regulation of histone lysine methylation marks is pivotal for neuronal plasticity and complex behaviors, we hypothesized that *L3mbtl1* null mutant mice will show alterations in a broad range of emotional and cognitive behaviors, in comparison to *L3mbtl1+/+* control mice from the same colony. Of note, *L3mbtl1-/-* mice have normal life expectancy, and show a subtle, but significant, reduction in body weight compared to controls. However, in line with a previous study exploring the interrelation between body weight and isolation-induced stress and emotional functions in mice[53], the subtle, ~ 10% reduction in body weight in the mutant mice of our study is unlikely to account for the mild behavioral phenotypes that define these animals. Furthermore, the association between body weight and cognitive and emotional functions is complex, with the same behavioral phenotype, for example increased anxiety, reported both in mice with decreased [54] or increased [55] body weight. The *L3mbtl1-/-* cohort of the present study exhibited significantly decreased anxiety in the light-dark box paradigm, and after social isolation as stressor, in the open field test. However, this was not associated with generalized changes in anxiety and depression-related behaviors, because despair-related immobility scores in the forced swim and tail suspension tests, and performance in the elevated plus maze as an additional measure for anxiety, were not consistently different from controls. We note that immobility times in behavioral despair paradigm were reduced in some cohorts of *L3mbtl1-/-* mutant mice (Fig 1B), but such an effect was not observed in another set of animals in which genotype was examined together with housing conditions (group- versus single housed) (Fig 2B). Similarly, shock-induced retention was maintained after *L3mbtl1* deletion in the fear conditioning paradigm, despite the significant decrease in freezing times during the training sessions in null mutant mice (Fig 1D). Moreover, spatial working memory was not significantly different between genotypes both at baseline and after social isolation stress (Fig 2D). Locomotor activity in a novel environment (Figs 1A and 2A) and in the context of acute changes in dopaminergic signaling (Fig 3B) showed a subtle decrease in the mutants, but this effect was variable and not consistently observed across stressed and non-stressed conditions (Fig 3B).

Taken together, our findings demonstrate that *L3mbtl1* loss causes subtle changes in behavior in some but not all of the tests applied. Importantly, lower anxiety scores were evident in at least two independent paradigms (light-dark box and open field test after social isolation). These findings are interesting given that other regulators of histone lysine methylation had been previously implicated to influence depression- and anxiety-related phenotypes. For example, mice with transgene-mediated overexpression of KMT1E/SETDB1/ESET histone H3-lysine 9 methyltransferase show robust antidepressant-like phenotypes in behavioral paradigms for despair, learned helplessness and anhedonia (defined as the inability to experience pleasure)[25]. Conversely, mice with a null mutation of *Kap1*, encoding the KMT1E-binding partner KRAB-associated protein 1, display elevated levels of anxiety together with impairments in learning and memory [56]. Notably, in mice exposed to social defeat, which is a strong stressor linked to the emergence of depression-related phenotypes, the H3K9-specific demethylase *Kdm3a* is subject to repressive chromatin remodeling in the ventral striatum, a key structure in the neuronal circuitry controlling emotional and affective behaviors [57]. In some studies, global changes in hippocampal histone methylation have been linked to depression-related phenotypes [58,59]. There is also evidence that non-selective monoamine oxidase inhibitors acting as antidepressants could potentially inhibit LSD1 type histone demethylases (which contain an amine oxidase domain) even at within the therapeutic range of these drugs [60]. Therefore, the present findings implicating *L3mbtl1* in the control of mood and behavior warrant further investigation to explore the therapeutic potential of this histone methyl-reader protein in psychiatric disorders. For example, it will be interesting to explore chromatin sites and target genes regulated by *L3mbtl1* and other MBT proteins in preclinical models for anxiety and depression.
